# Shifts in the pelagic ammonia-oxidizing microbial communities along the eutrophic estuary of Yong River in Ningbo City, China

**DOI:** 10.3389/fmicb.2015.01180

**Published:** 2015-10-27

**Authors:** Qiufang Zhang, Fangyuan Tang, Yangjing Zhou, Jirong Xu, Heping Chen, Mingkuang Wang, Hendrikus J. Laanbroek

**Affiliations:** ^1^Faculty of Architectural Civil Engineering and Environment, Ningbo UniversityNingbo, China; ^2^Department of Microbial Wetland Ecology, Netherlands Institute of Ecology (NIOO-KNAW)Wageningen, Netherlands; ^3^Institute of Environmental Biology, Utrecht UniversityUtrecht, Netherlands

**Keywords:** ammonia-oxidizing archaea (AOA), ammonia-oxidizing bacteria (AOB), eutrophic status, salinity, surface water

## Abstract

Aerobic ammonia oxidation plays a key role in the nitrogen cycle, and the diversity of the responsible microorganisms is regulated by environmental factors. Abundance and composition of ammonia-oxidizing archaea (AOA) and ammonia-oxidizing bacteria (AOB) were investigated in the surface waters along an environmental gradient of the Yong River in Ningbo, East China. Water samples were collected from three pelagic zones: (1) freshwaters in the urban canals of Ningbo, (2) brackish waters in the downstream Yong River, and (3) coastal marine water of Hangzhou Bay. Shifts in activity and diversity of the ammonia-oxidizing microorganisms occurred simultaneously with changes in environmental factors, among which salinity and the availabilities of ammonium and oxygen. The AOA abundance was always higher than that of AOB and was related to the ammonia oxidation activity. The ratios of AOA/AOB in the brackish and marine waters were significantly higher than those found in freshwaters. Both AOA and AOB showed similar community compositions in brackish and marine waters, but only 31 and 35% similarity, respectively, between these waters and the urban inland freshwaters. Most of AOA-*amoA* sequences from freshwater were affiliated with sequences obtained from terrestrial environments and those collected from brackish and coastal areas were ubiquitous in marine, coastal, and terrestrial ecosystems. All AOB from freshwaters belonged to *Nitrosomonas*, and the AOB from brackish and marine waters mainly belonged to *Nitrosospira*.

## Introduction

Excessive inorganic nitrogen is often the cause of eutrophication, which is a key important factor contributing to habitat variation and to catastrophic algal bloom expansion in aquatic ecosystems (Glibert et al., 2005). At the same time, complex relations between ammonia oxidizers and canonical denitrifiers are supposed to contribute to about 70% of the global N_2_O emissions, which has detrimental effects on global warming and on the Earth’s ozone layer (IPCC, 2007; Hatzenpichler, 2012). Being the first step of a coupled nitrification/denitrification process, which enables the removal of a large part of anthropogenically produced reactive nitrogen, aerobic ammonia oxidation is of pivotal significance in aquatic ecosystems (Seitzinger, 1988; Francis et al., 2007). Ammonia oxidation is controlled by the enzyme ammonia monooxygenase, which is partly encoded by the *amoA* gene (Rotthauwe et al., 1997; Kowalchuk and Stephen, 2001). Hence, *amoA* gene is a useful molecular marker for ammonia-oxidizing microorganisms in the environment (Francis et al., 2005, 2007).

Besides ammonia-oxidizing bacteria (AOB), ammonia-oxidizing archaea (AOA) are also potential contributors to nitrification (Francis et al., 2007; Prosser and Nicol, 2012). Previous studies have shown that the AOA and AOB communities were sensitive to many environmental factors (Erguder et al., 2009; Fernàndez-Guerra and Casamayor, 2012; Hatzenpichler, 2012; Cao et al., 2013). Estuaries are considered as major players in the transformation and removal of reactive nitrogen before it reaches the sea (Seitzinger et al., 2006). The steep physicochemical gradient in an estuary provides an ideal natural laboratory for studying ammonia-oxidizing archaea and bacteria (Bernhard and Bollmann, 2010; Santoro, 2010). Many worldwide studies in estuaries have reported that spatial variation in environmental factors drive shifts in abundance and community composition of ammonia-oxidizing microorganisms in sediments, i.e., in the Sacramento San Joaquin Delta (Damashek et al., 2015), the Scheldt estuary (Sahan and Muyzer, 2008), the Chesapeake Bay (Francis et al., 2003), the Plum Island Sound (Bernhard et al., 2005, 2007, 2010), the Ythan Estuary (Freitag et al., 2006), the Tokyo Bay (Urakawa et al., 2006), the Bahía del Tóbari estuary (Beman and Francis, 2006), the San Francisco Bay (Mosier and Francis, 2008), the Huntington Beach (Santoro et al., 2008), the Barn Island salt marsh (Moin et al., 2009), the Jiaozhou Bay (Dang et al., 2010), the Pearl River estuary (Cao et al., 2011; Jin et al., 2011; Xie et al., 2014), the Elkhorn Slough estuary (Wankel et al., 2011), and the Qiantang River (Liu et al., 2013). The studies of de Bie et al. (2001) and of Bollmann and Laanbroek (2002) are the only studies performed in the water column of an estuary, i.e., the Scheldt estuary. The series of studies have indicated that environmental factors such as salinity, trophic status, oxygen and temperature are likely to be potential selective factors shaping ammonia-oxidizing microbial communities.

The Yong River that passes through Ningbo City in eastern China is a typical example of a eutrophic coastal river system. Due to a limited runoff in relation to the tidal flows, pollutants emitted by anthropogenic activities have caused serious eutrophication problem in the aquatic ecosystem of Ningbo City, because most canals of the water system are constrained by dams built to defend against high tides, before they converge into the mainstream Yong River (Zhao and Yang, 2009). Therefore, to relieve problems with reactive nitrogen in the urban ecosystem, more should be known about the composition of the ammonia-oxidizing microbial communities and the dynamics of the ammonia oxidation process in this water system.

We hypothesized that the activity, abundance and species composition of AOA and AOB in the surface waters of the Yong River will be affected by the physicochemical water properties at the different zones of the estuary. The characteristics of the ammonia-oxidizing microorganisms were investigated in fresh, brackish and marine waters. The composition and abundance of *amoA* genes for both AOB and AOA in surface waters were compared between the fresh, brackish and marine areas. The relationship between ammonia oxidizers diversity and ammonia oxidation rate was also determined.

## Materials and Methods

### Sampling Description and Water Properties

**FIGURE 1:**
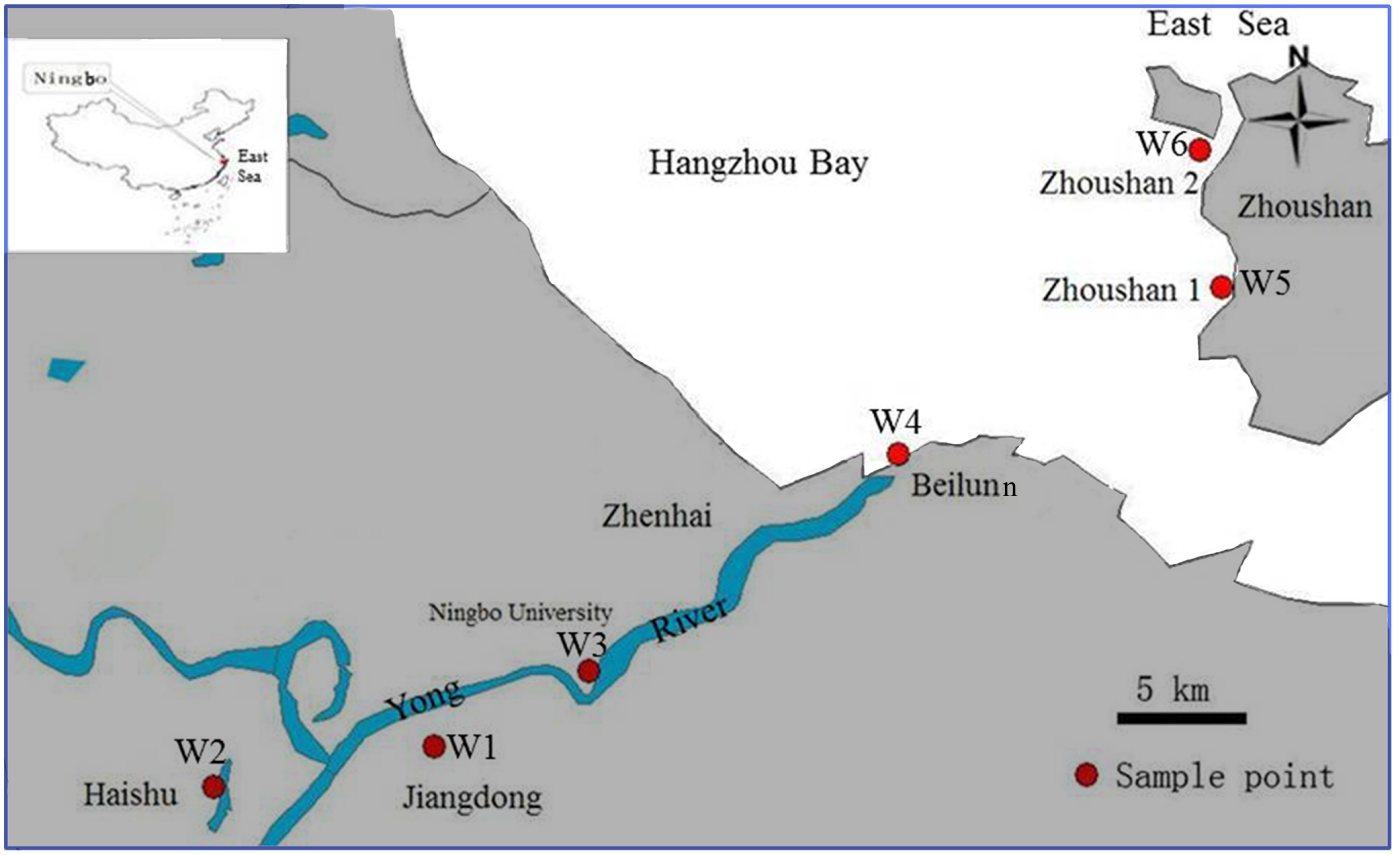
**Locations of the sampling sites in the Ningbo City urban canals (W1 and W2), in the Yong River estuary (W3 and W4) and in the Hangzhou Bay (W5 and W6)**.

As a coastal river system, the Yong River consists mainly of the Yao River, the Fenghua River, and the Yong River. The mainstream Yong River is about 26 km in length, from Sanjiangkou to Zhenhai, which is the entrance to the East China Sea (**Figure 1**). The runoff of the Yong River is only 5–23% of the tidal flows (Zhao and Yang, 2009). Sampling zones were divided into three geographic regions along the Yong River spanning freshwater, brackish water, and marine coastal water sites: (1) two freshwater sites in urban canals at Jiangdong (W1; N29°51′50.7″, E121°35′43.6″) and Haishu (W2; N29°53′55.4″, E121°31′45.9″), respectively; (2) two brackish sites near Ningbo University (W3; N29°54′32.1″, E121°37′35.4″) and in the Beilun estuary (W4; N29°58′14.2″, E121°46′36.8″), respectively; and (3) two coastal water sites near the island of Zhoushan (W5; N29°59′04.2″, E121°50′52.3″) and (W6; N30°02′08.1″, E121°50′ 29.4″) both in the Hangzhou Bay remote from residential and large-scale industrial areas and hence waste and freshwater were absent. Triplicate water samples were collected within a 5 m radius at each sampling site from a depth of 30 cm under the water surface. Water sampling was carried out at low tide on November 23, 2012.

The water parameters of salinity, pH, and dissolved oxygen (DO) were determined *in situ* by a portable meter (Hach Company, Loveland, CO, USA), and the *in situ* temperature was about 12°C. The triplicate water samples were separated in two portions after removal of larger particles and transferred into sterilized bottles; one portion was placed on ice in a cool box for extracting DNA and the other portion was kept at 4°C for determining water properties and potential ammonia oxidation activity (PAA). Samples were transported to the laboratory within 2 h. The portion of 500 mL waters kept at 0°C were filtered through 0.22 μm pore-sized filters (47 mm diameter, Durapore GVWP, Millipore) to collect all microorganisms. The filters were stored and frozen at -80°C until further analysis. The ammonium (NH_4_^+^) and nitrite (NO_2_^-^) concentrations were determined by a flow injection analyzer (Lachat-QC8500, Lachat Instruments, Loveland, CO, USA), total nitrogen (TN) and nitrate (NO_3_^-^) were also determined by the flow injection analyzer after alkaline persulfate digestion and cadmium-copper reduction, respectively. Total organic matter (TOC) was determined by a total organic carbon analyzer (TOC-VCPH, Shimadzu).

### Potential Ammonia Oxidation Rates

Potential ammonia oxidation activity of water samples was measured according to a previously described method with minor modification (Kasuga et al., 2010). Samples were taken at 0, 6, 12, 24, and 48 h for determining the ammonium concentrations. Generally, ammonium consumption accelerated after 6 h, but slowed down again after 12 h of incubation. The decrease in ammonium concentrations between 0 and 6 h was used to determine the potential activity as this reflected mostly the potential ammonium consumption rate in the water column of the estuary. The rate determinations were carried out by incubating 400 mL in the dark at 25°C at 100 rpm. Inorganic medium per liter of water was composed of (NH_4_)_2_SO_4_ 0.18 mM, MgSO_4_⋅7H_2_O 0.81 mM, CaCl_2_ 0.18 mM, K_2_HPO_4_ 0.76 mM, NaH_2_PO_4_ 1.30 mM, Fe-EDTA 0.02 mM, CaCO_3_ 2.00 mM, Na_2_MoO_4_⋅2H_2_O 4 nM, MnCl_2_⋅4H_2_O 10 nM, CoCl_2_⋅6H_2_O 8 nM, CuSO_4_⋅5H_2_O 80 nM and ZnSO_4_⋅7H_2_O 300 nM. The ammonium concentrations were determined according to the method described above.

### DNA Extraction

Extraction of the nucleic acids from the filters containing the microorganisms was performed using the Fast DNA^®^ spin kit for soil (MP Biomedicals, LLC, Illkirch, France). The filters were cut into small pieces and transferred into the Lysing Matrix E tubes containing a matrix designed for the lysis of most of the cell types, using ethanol flamed forceps and surgical scissors. The mixture was homogenized in a FastPrep^®^-24 (Bio 101, CA) for 15 s at 4 m⋅s^-1^ (Sekiguchi et al., 2002). The extraction procedure was performed as described in the manufacturer’s instructions. DNA extracts were suspended in 100 μL of DES solution (Qbiogene, Vista, CA, USA) and quantified by a Nanodrop spectrophotometer. The DNA extracts were stored frozen at -80°C until further analysis.

### PCR-DGGE Analysis

CrenamoA23f/CrenamoA616r (Tourna et al., 2008) and amoA1F-GC/amoA2R (Rotthauwe et al., 1997) were primers for the archaeal and bacterial *amoA* genes, respectively (**Table 1**). Amplifications were performed for AOA and AOB in 50 μL reaction mixtures including 1× PCR buffer, 3.0 mM MgCl_2_, 400 μM of each dNTP, 2.5 U Taq DNA polymerase (Takara, Dalian, China) and 0.2 mg mL^-1^ bovine serum albumin (BSA) plus 0.2 mM of each primer, 2 and 4 μL genomic DNA (80–120 ng μL^-1^) were added as template for AOA and AOB, respectively.

**Table 1:** Primers, qPCR and PCR-DGGE conditions used.

Target group	Primer	Sequence (5′–3′)	Amplicon length (bp)	Thermal profile for real-time PCR	Thermal profile for PCR and DGGE conditions	Reference
AOA-*amoA*	CrenamoA23f	ATGGTCTGGCTWAGACG	629	50°C 2 min, 95°C 10 min, followed by 40 cycles of 95°C 15 s, 58°C1 min, and fluorescence was read during each cycle at 83°C	95°C 5 min, followed by 10 cycles of 94°C 30 s, 55°C 30 s and 72°C 1 min, and 25 cycles of 92°C 30 s, 55°C 30 s and 72°C 1 min, and 72°C 10 min; 8% (w/v) polyacrylamide [acrylamide-bisacrylamide (37.5:1)] gels containing denaturing gradients of 15–55%. Electrophoreses were run at 95 V for 14 h.	Tourna et al., 2008
	CrenamoA 616r	GCCATCCATCTGTATGTCCA				
AOB-*amoA*	*amoA*-1F *amoA*-1F^a^	GGGGTTTCTACTGGTGGT	491	50°C 2 min, 95°C 10 min, followed by 40 cycles of 95°C 15s, 60°C 1 min, and fluorescence was read during each cycle at 83°C	94°C 4 min, followed by 20 cycles: 94°C 1 min, 61–52°C touchdown (-0.5°C 1 min per cycle), and 72°C 1 min, and 17 cycles: 94°C 1 min, 52°C 1 min, and 72°C 1 min, and 72°C 10 min. 6% (w/v) polyacrylamide gels containing denaturing gradients of 40–60%. Electrophoreses were run at 80 V for 12 h.	Rotthauwe et al., 1997
	*amoA*-2R	CCCCTCKGSAAAGCCTTCTTC[K = G or T, S = G or C]				

*^a^GC clamp (5′-CCGCCGCGCGGCGGGCGGGGCGGGGGCACGGGG-3′) was attached to the 5′ end of primer amoA-1F.*

DGGE analyses were carried out using a Dcode Universal Mutation Detection System (Bio-Rad Laboratories, Hercules, CA, USA) under the conditions presented in **Table 1**. 8 μL of the PCR products of either AOA-*amoA* or AOB-*amoA* genes were loaded into polyacrylamide gel wells. The gels were stained with 1:10,000 SYBRGreen I (Sigma, Taufkirchen, Germany) for 30 min and scanned by a GelDoc XR (BIO-RAD, Hercules, CA, USA).

### Cloning and Sequencing

Selected DGGE bands were excised for cloning and sequencing according to the method described in a previous study (Zhang et al., 2011). Excised bands were crushed and immersed overnight at 4°C in 30 μL of sterilized water to release DNA, and subsequently amplified with the primers CrenamoA 23f/CrenamoA616r (Tourna et al., 2008) and amoA1F/amoA2R (Rotthauwe et al., 1997), respectively. PCR products were purified and linked into the PMD^TM^ 19-T Vector (TaKaRa Code: D102A, Dalian). The resulting ligation mixture was transformed into *Escherichia coli* DH5α competent cells following the instructions of the manufacturer. Positive clones were amplified using the above-mentioned primers with a GC clamp and cross-checked by DGGE for migration behavior and sent to Invitrogen (Shanghai, China) for sequencing.

### Real-time PCR (qPCR)

qPCR assays of *amoA* gene of all samples were performed in triplicates using an Eppendorf Mastercycler^®^ ep realplex2 machine (Eppendorf, Hamburg, Germany). Each reaction was performed in a 25 μL volume containing 1 μL of DNA extract (80–120 ng μL^-1^) as template, 0.2 mg mL^-1^ BSA, 0.4 mM of each primer and 12.5 μL Premix Ex taq^TM^ (Takara, Dalian). A total of 40 cycles was run with the procedure and primers listed in **Table 1**. Product specificity was confirmed by melting curve analysis and visualization on 1.2% agarose gels. Specific products were observed at the expected sizes of ca. 491 and 629 bp for the bacterial and archaeal *amoA* genes, respectively.

Standard curves for qPCR were developed as previously described (Zhang et al., 2011). Archaeal and bacterial *amoA* genes fragments were cloned as described in the section of cloning and sequencing. The qPCR standard DNA was amplified from representative plasmids containing AOA-*amoA* or AOB-*amoA* gene linearized by EcoR I, and then purified by a Purification Kit (Tiangen Biotech Co., China). The concentration of the purified DNA was measured with a spectrophotometer (Nanodrop). Ten-fold serial dilutions of a known copy number of the *amoA* gene were generated to produce the standard curve over seven orders of magnitude (from 2.23 × 10^2^ to 2.23 × 10^8^ copies for AOA-*amoA*, and from 6.51 × 10^2^ to 6.51 × 10^8^ copies for AOB-*amoA*) per assay. High amplification efficiencies of 95.0–93.6% were obtained for archaeal and bacterial *amoA* quantification with *R*^2^ values 0.990 and 1.000, slopes were -3.35 and -3.38, respectively. A negative control without template DNA (NTC) was present in every qPCR assay. No evident inhibition was found with any of the samples. Inhibition of DNA extraction was tested using a previously described method (Dumonceaux et al., 2006). No significant inhibition was observed at any dilution (1–120 ng μL^-1^ DNA extract).

### Statistical Analysis

Based on DGGE banding patterns, similarity of water samples was elucidated with an unweighted pair group method with mathematical averages (UPGMA) using the software package Quantity One (Bio-Rad Laboratories, Hercules, CA, USA). The diversity indices of Shannon (*H*), Evenness (*J*) and Simpson (*D*) were calculated for DGGE band pattern of *amoA* genes with the PRIMER5.0 software. The one-way ANOVA and Pearson’s coefficient analysis were carried out with SPSS software (version 17.0; SPSS Statistics Inc., Chicago, IL, USA). To explore which environmental factors contributed to the changes among sites, principal-component analysis (PCA) was performed with PAST ver. 2.17c (Hammer et al., 2001), based on the water properties, including water salinity, pH, DO, TN, ammonium (NH_4_^+^-N), nitrite (NO_2_^-^–N), nitrate (NO_3_^-^–N) and total organic carbon (TOC) for all six stations.

Sequences were compared with GenBank database using BLAST (http://www.ncbi.nlm.nih.gov/BLAST/), and the closest matches were included in the alignment and phylogenetic analysis. The neighbor-joining trees were constructed using MEGA 4 and bootstrapped 1,000 times to calculate linear distances, and alignments were edited using the Bioedit sequence alignment Editor 7.0.5.2 (Tamura et al., 2007).

### Sequence Accession Numbers

All sequences from DGGE bands have been deposited in the EMBL nucleotide sequence database with accession numbers from LN845817 to LN845837 for the AOA-*amoA* genes, and from LN845795 to LN845813 for the AOB-*amoA* genes.

## Results

### Water Properties and Potential Nitrification Rates

Water properties varied among the different sampling sites (**Table 2**). Water salinity ranged from 0.20 to 24.60%. The highest salinities were found at Zhoushan 1 and 2 (W5 and W6), followed by Beilun estuary (W4: 7.60%) and Yong River next to Ningbo University (W3: 4.07%), and the lowest value of 0.20% was observed at Jiangdong (W1) and Haishu (W2). The salinities in the coastal waters at W5 and W6 were significantly more saline than those at the other sites, and the salinities in the Yong River downstream of W3 and W4 were also significantly higher than those of the urban canals at W1 and W2. Water pH at the urban canal sites W1 and W2 was lower than at W3, W5, and W6, but similar to the pH of W4. DO concentrations at W1 and W2 were significantly lower than at other sites, whereas the highest DO was found at W4 in the Beilun estuary. The values of TN, NH_4_^+^-N, NO_2_^-^-N, NO_3_^-^-N and TOC measured at W1, W2, and W3 were significantly lower than those determined at W4, W5, and W6. According to the “Environmental quality standard for surface water” (GB3838-2002, China; Zhao and Yang, 2009), water samples collected at the urban canal sites W1 and W2 were hyper eutrophic.

**Table 2:** Average water properties at the freshwater, brackish and marine sampling sites.

Sampling region	Salinity (%)	pH	DO (mg L^-1^)	TN (mg L^-1^)	NH_4_^+^-N (mg L^-1^)	NO_2_^-^-N (mg L^-1^)	NO_3_^-^-N (mg L^-1^)	TOC (mg L^-1^)	PAA mg L^-1^ h^-1^
Jiangdong (W1)	0.20 e^a^	7.22 b	4.15 e	13.17 a	9.02 b	2.19 b	3.45 c	16.71 a	0.009 c
Haishu (W2)	0.20 e	7.13 b	3.56 e	13.28 a	9.85 a	3.25 a	4.85 a	10.76 b	0.018b c
Yong River next to Ningbo University (W3)	4.07 d	7.70 a	8.73 c	4.58 b	1.49 c	0.08 c	4.44 b	9.98 c	0.020b c
Beilun estuary (W4)	7.60 c	7.20 b	11.53 a	2.37 cd	0.14 de	0.02 c	1.60 e	6.53 e	0.026 b
Zhoushan1 (W5)	23.50 b	7.56 a	9.43 b	2.16 d	0.03 e	0.01 c	1.20 f	8.27 d	0.030 b
Zhoushan2 (W6)	24.60 a	7.70 a	7.47 d	2.58 c	0.25 d	0.03 c	1.69 d	8.50 d	0.046 a

*^a^Values within the same column followed by the same letter do not differ at *P* < 0.05.*

**FIGURE 2:**
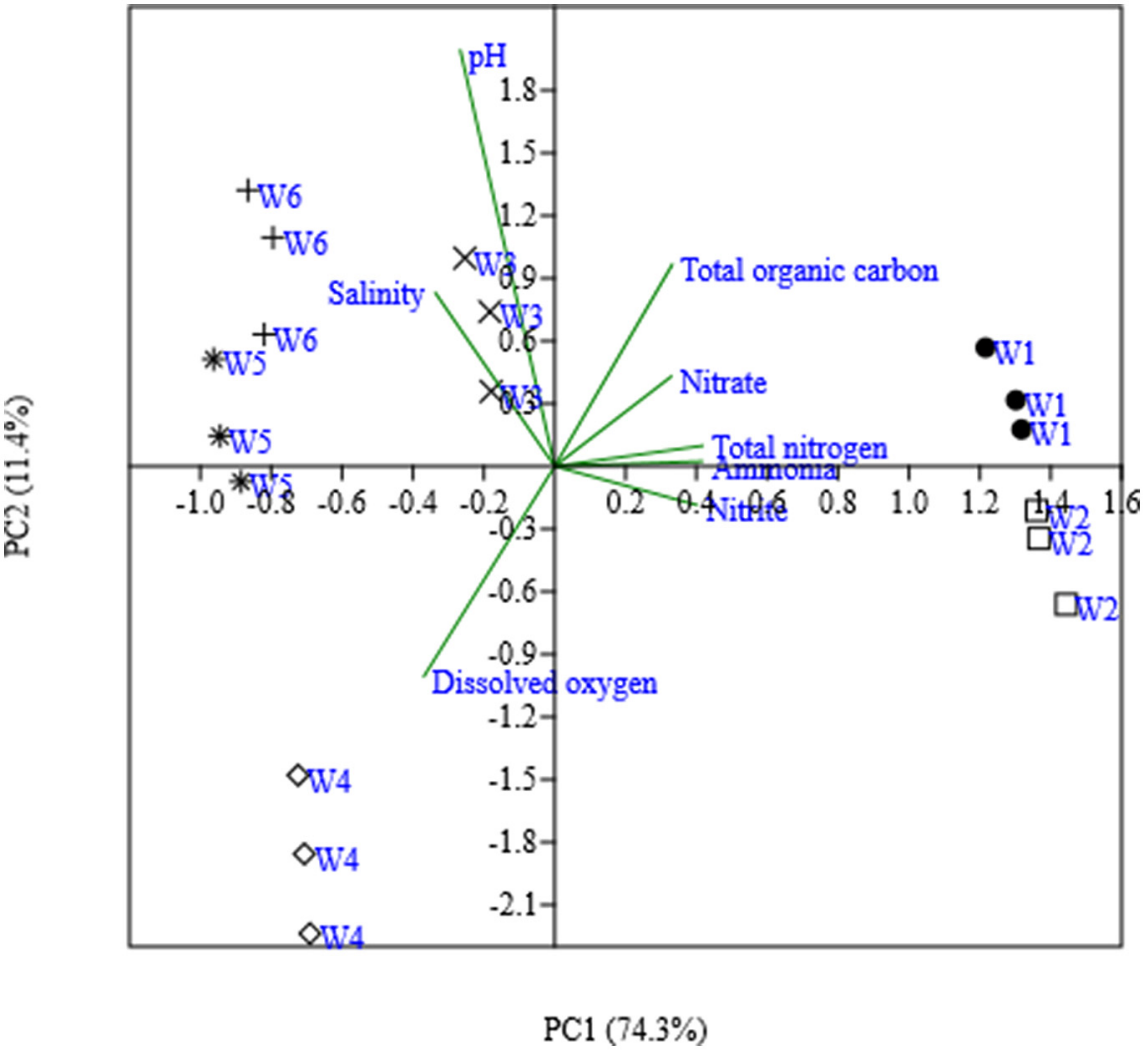
**Ordinate plot from principal-component analysis (PCA) base on water properties from six different sites.** The values in parentheses are percentages of the total variances of PCA derived from water parameters.

A Principal Component Analyses was performed on the water properties at the different sampling locations. The first two principal components explained a large amount (85.7%) of the variation in the environmental factors and strong geographical clusters were formed (**Figure 2**). The first principal component (PC 1), which explained 74.3% of the variation, separated DO, salinity, and pH from the other parameters (TN, NH_4_^+^-N, NO_2_^-^-N, NO_3_^-^-N, and TOC), while the second principal component (PC 2) explaining 11.4% of the variation separated mainly DO from pH, salinity and TOC. The nitrogen-related data (e.g., nitrate, nitrite, ammonium, and TN) were not separated by these two principal components. The freshwater sampling locations W1 and W2 clustered together and were mostly characterized by higher concentrations of TN, NH_4_^+^-N, NO_2_^-^-N, NO_3_^-^-N, and TOC. The marine water locations W5 and W6 grouped also together at higher values of salinity, and pH. The brackish water sampling locations W3 and W4 did not cluster together. W3 was positioned between the freshwater and marine locations, whereas W4 separated from the all other locations by high oxygen concentrations.

Potential ammonia oxidation activities were also different among the fresh, brackish and marine water sites (**Table 2**). PAA was higher at the brackish and marine sites (W3, W4, W5, and W6) than at the freshwater sites (W1 and W2). PAA was positively correlated with DO (*R* = 0.539, *n* = 18, *P* < 0.05) and salinity (*R* = 0.768, *n* = 18, *P* < 0.01). However, PAA was negatively related to TN (*R* = -0.684, *n* = 18, *P* < 0.01), NO_3_^-^-N (*R* = -0.659, *n* = 18, *P* < 0.01), NO_2_^-^-N (*R* = -0.572, *n* = 18, *P* < 0.05), NH_4_^+^-N (*R* = -0.655, *n* = 18, *P* < 0.01) and TOC (*R* = -0.636, *n* = 18, *P* < 0.01). There was no significant correlation between PAA and pH (*R* = 0.439, *n* = 18, *P* = 0.068).

### Spatial Variation in *amoA* Abundance

The abundance of AOA-*amoA* and AOB-*amoA* genes was different in the three water areas (**Table 3**). For AOA, *amoA* gene copy numbers per liter of water varied between 2.31 × 10^6^ and 7.33 × 10^7^. The lowest number of AOA-*amoA* was found in water of the Yong River next to Ningbo University (W3). AOA-*amoA* gene numbers at W4, W5, and W6 were significantly higher than those observed at W1, W2, and W3. AOA-*amoA* gene copy numbers positively related to salinity (*R* = 0.818, *n* = 18, *P* < 0.01), DO (*R* = 0.716, *n* = 18, *P* < 0.01), and PAA (*R* = 0.697, *n* = 18, *P* < 0.01), respectively. AOA-*amoA* gene copy numbers were negatively correlated with TN (*R* = -0.794, *n* = 18, *P* < 0.01), NO_3_^-^-N (*R* = -0.941, *n* = 18, *P* < 0.01), NO_2_^-^-N (*R* = -0.698, *n* = 18, *P* < 0.01), NH_4_^+^-N (*R* = -0.810, *n* = 18, *P* < 0.01) and TOC (*R* = -0.732, *n* = 18, *P* < 0.01), respectively.

**Table 3:** Copy numbers of AOA-*amoA* and AOB-*amoA* genes in surface waters.

	Copy numbers L^-1^ water		
Sampling region	AOA-*amoA*	AOB-*amoA*	AOA/AOB
W1	4.36 × 10^6^ ± 2.29 × 10^5^ d^a^	2.97 × 10^5^ ± 2.69 × 10^4^ d	14.74 ± 2.10 c
W2	2.96 × 10^6^ ± 7.70 × 10^3^ d	2.98 × 10^5^ ± 1.64 × 10^4^ d	9.98 ± 0.53 c
W3	2.31 × 10^6^ ± 2.40 × 10^4^ d	6.09 × 10^5^± 1.16 × 10^4^ a	3.80 ± 0.11 c
W4	7.33 × 10^7^ ± 2.10 × 10^6^ a	3.83 × 10^5^ ± 2.34 × 10^4^ c	191.88 ± 17.22 a
W5	6.57 × 10^7^ ± 2.30 × 10^6^ b	4.64 × 10^5^ ± 3.50 × 10^4^ b	143.71 ± 18.53 b
W6	5.75 × 10^7^ ± 1.74 × 10^6^ c	3.75 × 10^5^ ± 1.01 × 10^4^ c	153.43 ± 8.81 b

*^a^Mean ± SD (*n* = 3). Values within the same column followed by the same letter do not differ at *P* < 0.05.*

qPCR assays showed that bacterial *amoA* gene copy numbers per liter of water ranged from 2.97 × 10^5^ to 6.09 × 10^5^. The highest number of AOB-*amoA* was found in water of the Yong River next to Ningbo University (W3). AOB-*amoA* gene numbers in the brackish and marine areas (W3, W4, W5, and W6) were significantly higher than those observed in the freshwater sites W1 and W2. Positive correlations were found between AOB-*amoA* gene copy numbers and salinity (*R* = 0.895, *n* = 18, *P* < 0.01), pH (*R* = 0.515, *n* = 18, *P* < 0.01) and DO (*R* = 0.752, *n* = 18, *P* < 0.01), respectively, but AOB-*amoA* gene copy numbers negatively correlated with TN (*R* = -0.904, *n* = 18, *P* < 0.01), NO_2_^-^-N (*R* = -0.819, *n* = 18, *P* < 0.01), and NH_4_^+^-N (*R* = -0.883, *n* = 18, *P* < 0.01). No significant correlation was observed between AOB-*amoA* gene copy numbers and PAA (*R* = 0.326, *n* = 18, *P* = 0.187) and TOC (*R* = -0.360, *n* = 18, *P* = 0.142), respectively.

Ammonia-oxidizing archaea-*amoA* gene numbers were always more abundant than those of AOB in all water samples (**Table 3**). Especially, the ratios of AOA/AOB at the more marine sites (W4, W6, and W5) were much higher than those at the other sites.

### Composition of AOA-*amoA* Genotypes

**FIGURE 3:**
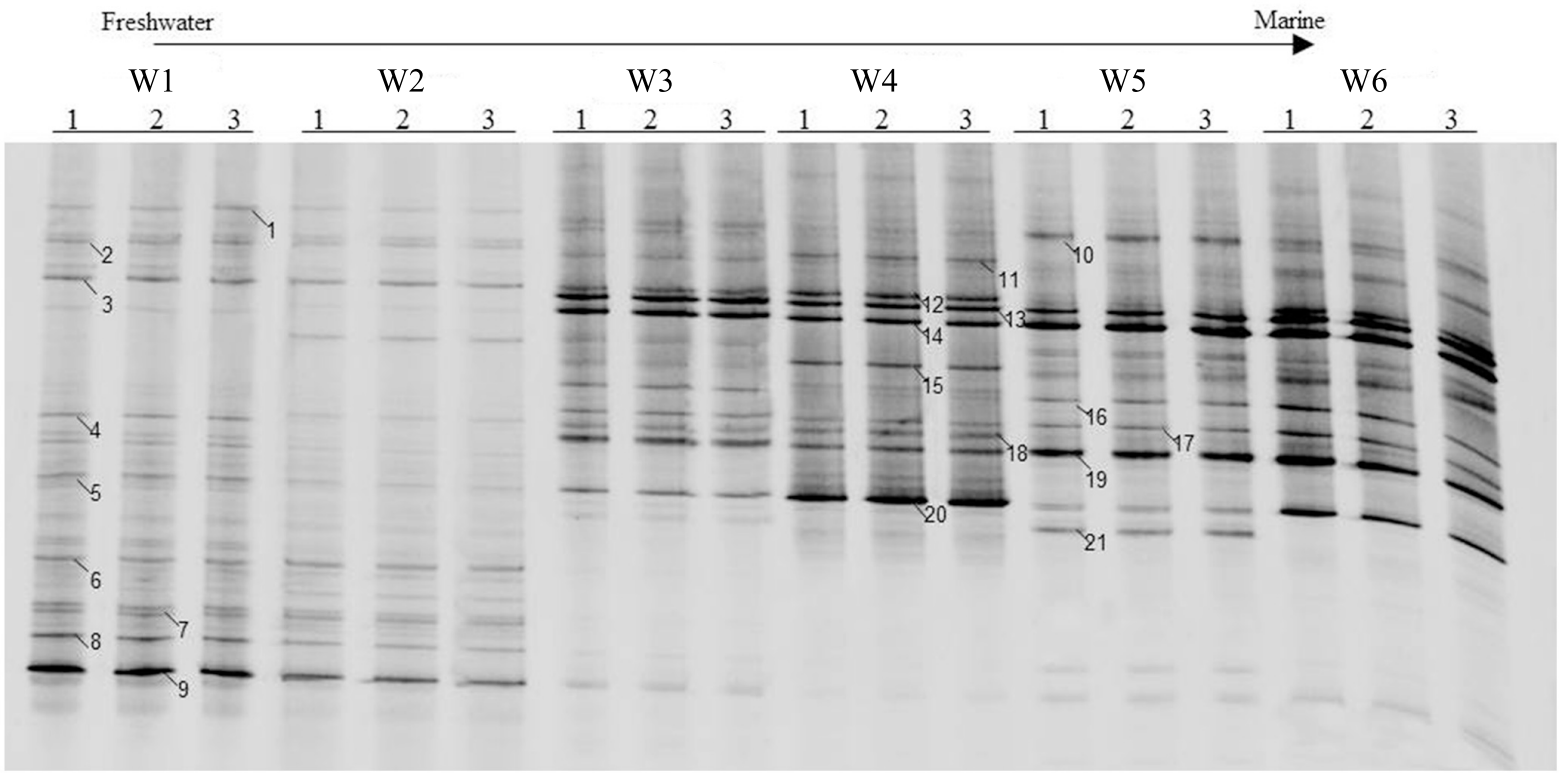
**Denaturing gradient gel electrophoresis profiles of archaeal *amoA* genes in surface water collected from the sampling sites W1, W2, W3, W4, W5, and W6 along the Yong River.** Bands used for sequencing and phylogenetic analysis are highlighted.

**FIGURE 4:**
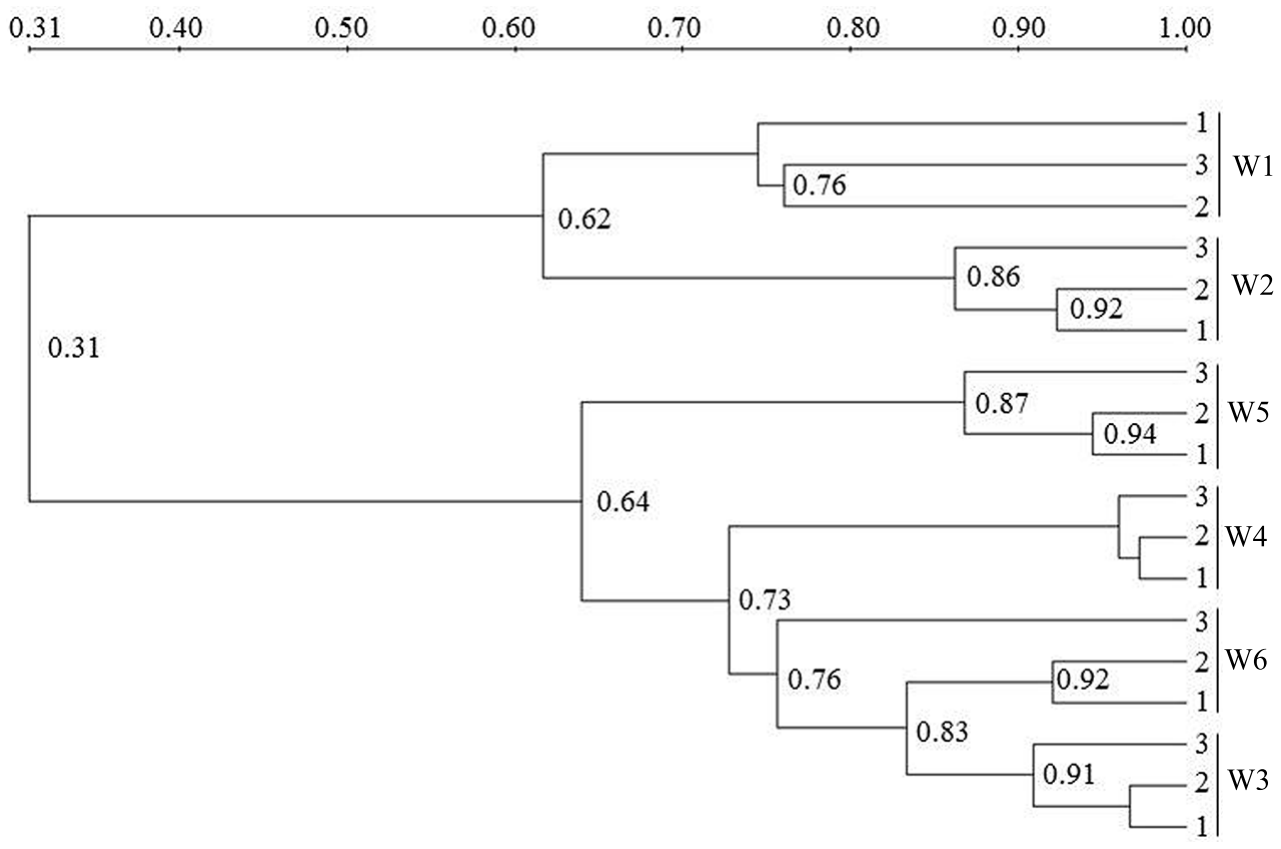
**Cluster analysis unweighted pair group method with mathematical averages (UPGMA) of ammonia-oxidizing archaea (AOA) community composition based on the DGGE band pattern in surface water from the different sampling sites W1, W2, W3, W4, W5, and W6.** The numbers in the figure indicate the clustering similarities.

The AOA community composition was analyzed by PCR-DGGE (**Figures 3** and **4**). The triplicate banding profiles of each sampling sites clustered together. The patterns of AOA at the freshwater sites W1 and W2 were distinct from those observed at the brackish sites W3 and W4, and at the coastal sites W5 and W6. The similarity between the freshwater sites and the other sites was only 31%, whereas one of the marine sites (W5) showed 64% similarity with a cluster of sites that was comprised of the other marine site (W6) and the brackish sampling sites (W3 and W4), which showed 76% mutual similarity.

The Shannon, Evenness and Simpson indices were used to establish the ammonia oxidizers diversity (**Table 4**). Of the AOA communities, the Shannon and Simpson indices were significantly higher at the freshwater site W1 than at all the other sites. In contrast to this difference in similarity indices, the Evenness index was significantly higher at the marine site W6 than at the other sites. The Shannon index was significantly and positively correlated with TN (*R* = 0.646, *n* = 18, *P* < 0.01), NO_2_^-^-N (*R* = 0.530, *n* = 18, *P* < 0.05) and NH_4_^+^-N (*R* = 0.622, *n* = 18, *P* < 0.01), but negatively with pH (*R* = -0.491, *n* = 18, *P* < 0.05) and DO (*R* = -0.552, *n* = 18, *P* < 0.05). A significantly positive correlation was found between the Evenness index and salinity (*R* = 0.498, *n* = 18, *P* < 0.05), and a negative correlation between this index and TN (*R* = -0.475, *n* = 18, *P* < 0.05) and NH_4_^+^-N (*R* = -0.475, *n* = 18, *P* < 0.05).

**Table 4:** Diversity properties of AOA and AOB communities in the water layer at the different sampling locations based on DGGE band pattern data.

Sampling region	AOA			AOB		
	Shannon (*H*)	Evenness (*E*)	Simpson (*D*)	Shannon (*H*)	Evenness (*E*)	Simpson (*D*)
W**l**	2.792 a^a^	0.891 b	0.916 a	1.040 d	0.946 a	0.633 d
W2	2.566 b	0.894 b	0.898 be	1.958 c	0.942 ab	0.847 c
W3	2.552 b	0.906 b	0.898 be	2.350 b	0.916 c	0.889 b
W4	2.516 b	0.903 b	0.905 be	2.400 b	0.927 be	0.899 ab
W5	2.440 b	0.895 b	0.895 c	2.480 a	0.916 c	0.9064 a
W6	2.552 b	0.930 a	0.911 ab	2.376 b	0.909 c	0.894 ab

*^a^Values within the same column followed by the same letter do not differ at *P* < 0.05.*

**FIGURE 5:**
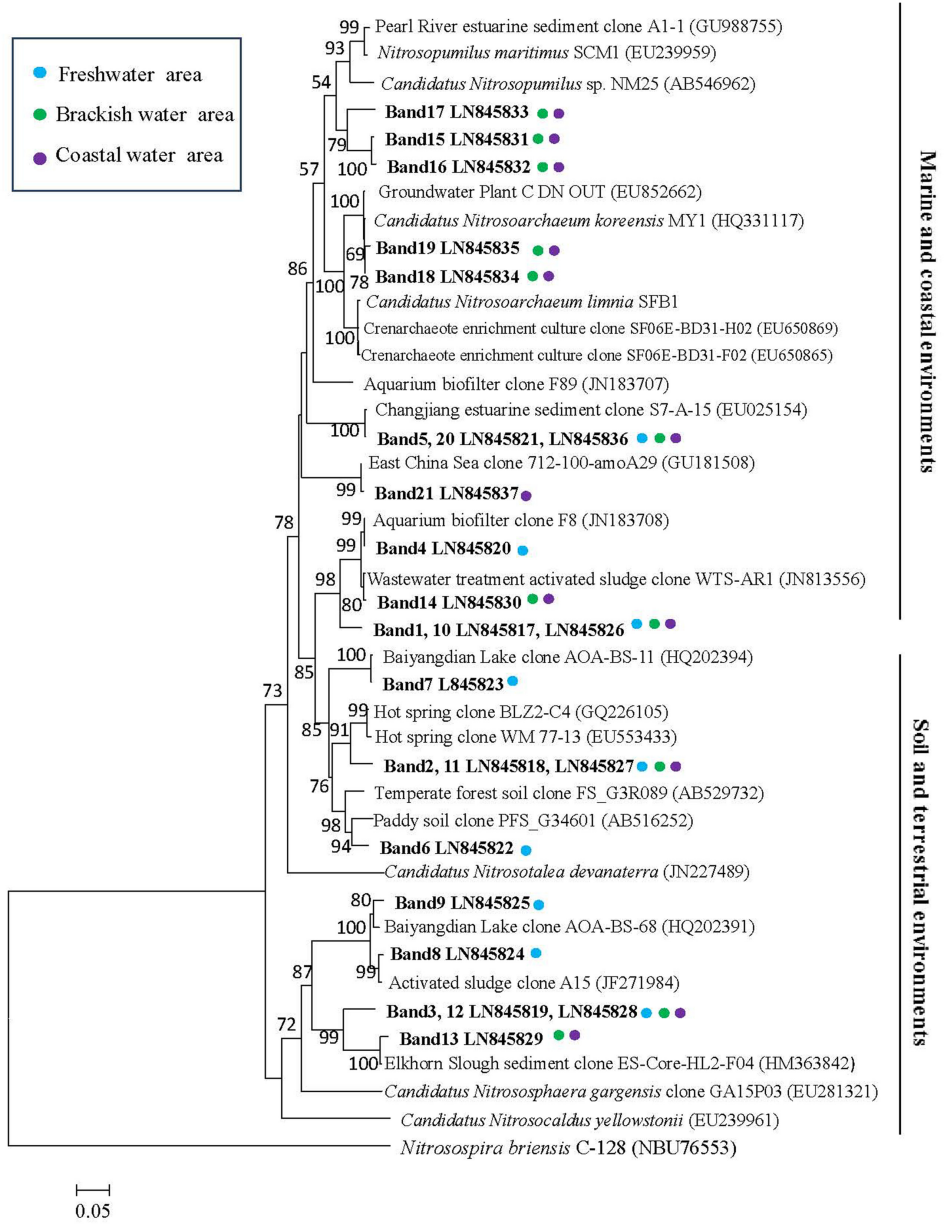
**Phylogenetic relationships among archaeal *amoA* gene sequences retrieved from surface waters.** Designation of the clones in bold includes the following information: accession number in the EMBL with DGGE band label. Colored points next to each retrieved gene sequences indicate the sampling station. Bootstrap values ≥ 50% (1000 neighbor-joining bootstraps) are shown at the branch points. The scale bar represents 5% estimated sequence divergence. The tree was rooted with *Nitrosospira briensis* C-128 (NBU76553).

Twenty-one bands of AOA-*amoA* gene were excised from the DGGE gels (**Figure 3**). The recovered sequences (629 bp) were used to build a phylogenetic tree (**Figure 5**). The sequences were affiliated with sequences found in marine and coastal habitats, and in terrestrial environments. Most of sequences collected from freshwater belong to the cluster from terrestrial environments, only the sequence of band 5 fell into the marine and coastal environments. The sequences from brackish and coastal areas were mainly grouped in the cluster from marine and coastal environments, which also included the cultures of *Candidatus Nitrosoarchaeum koreensis* MY1 and *Candidatus Nitrosoarchaeum limnia*, and two Crenarchaeote enrichment culture clones collected from the San Francisco Bay estuary. But some of the sequences from brackish and coastal areas were also distributed in the cluster from terrestrial environments, overlapping with those from freshwater samples.

### Composition of AOB-*amoA* Genotypes

**FIGURE 6:**
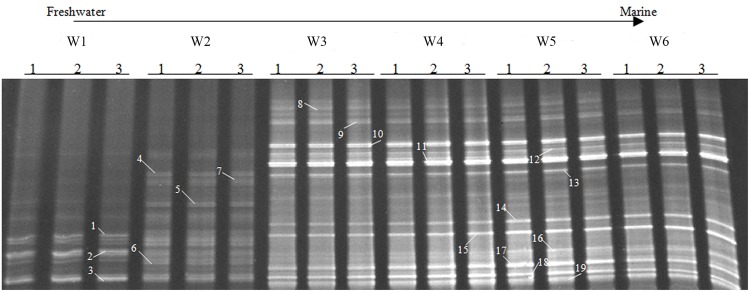
**Denaturing gradient gel electrophoresis profiles of bacterial *amoA* in surface water collected from the sampling sites W1, W2, W3, W4, W5, and W6 along the Yong River.** Bands used for sequencing and phylogenetic analysis are highlighted.

**FIGURE 7:**
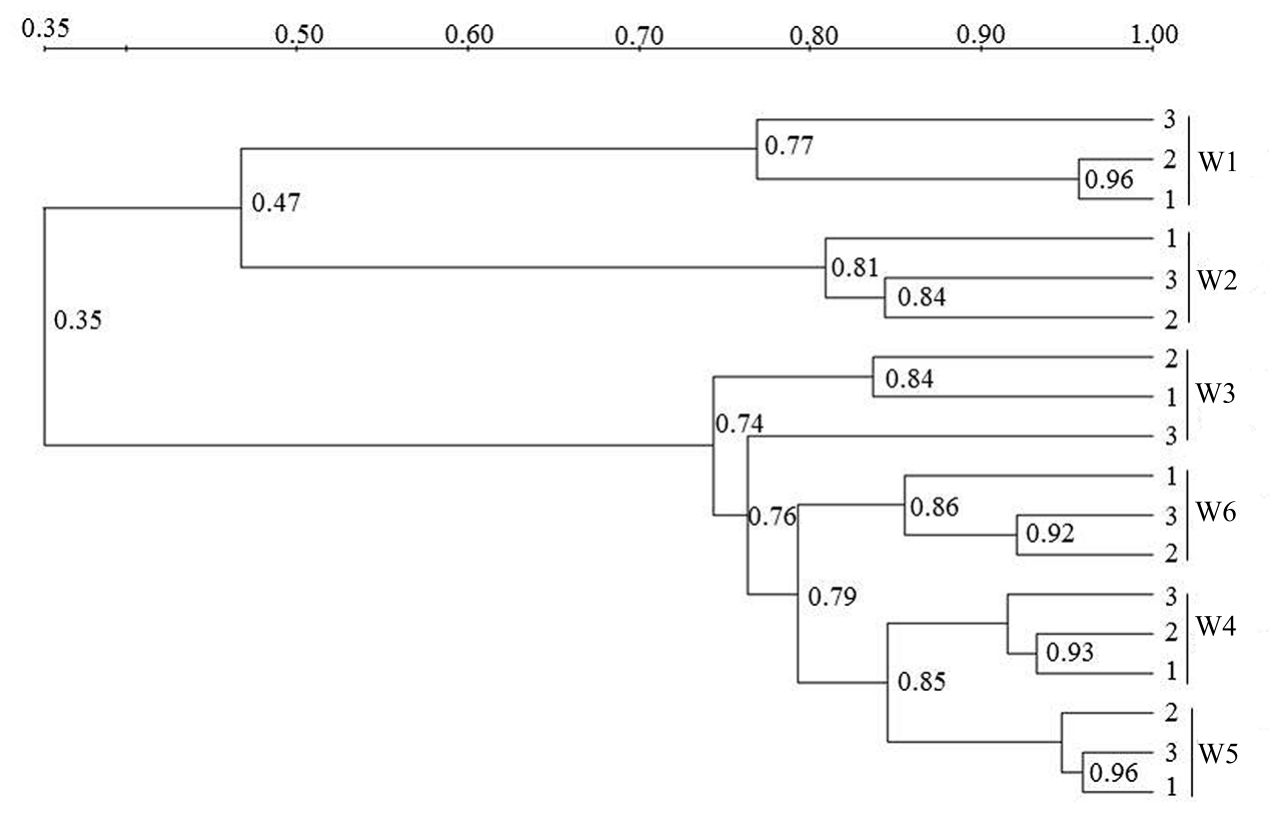
**Cluster analysis (UPGMA) of AOB community composition based on the DGGE band pattern in surface waters from the sampling sites W1, W2, W3, W4, W5, and W6.** The numbers in the figure indicate the clustering similarity.

Similar to the AOA patterns, the triplicate banding profiles of each sampling sites clustered together and the AOB community compositions in at the freshwater sites W1 and W2 were again distinct from those of the brackish and marine sites W3, W4, W5, and W6 (**Figures 6** and **7**). The similarity in DGGE patterns between the freshwater sites and the other sites was only 35%. With a similarity of 47%, both freshwater sites were more dissimilar from each other than the estuarine and marine sites that shared a similarity of higher than 74%.

The diversity and evenness indices of bacterial ammonia oxidizers diversity in freshwater (W1 and W2) were obviously different from brackish (W3 and W4) and coastal areas (W5 and W6; **Table 4**). In contrast to AOA, the AOB Shannon index was lower at W1 and W2 than at the other sites, and the Evenness index was higher at W1 and W2 compared with the other sites. The Simpson index was significantly lower at W1 than at the other sites. The Shannon was significantly and positively correlated with salinity (*R* = 0.606, *n* = 18, *P* < 0.01), pH (*R* = 0.504, *n* = 18, *P* < 0.05) and DO (*R* = 0.741, *n* = 18, *P* < 0.01), and negatively with TN (*R* = -0.846, *n* = 18, *P* < 0.01), NO_2_^-^-N (*R* = -0.699, *n* = 18, *P* < 0.01) and NH_4_^+^-N (*R* = -0.815, *n* = 18, *P* < 0.01), respectively. A significantly positive correlation was found between the Evenness index and salinity (*R* = 0.498, *n* = 18, *P* < 0.05), and a negative correlation between this index and TN (*R* = -0.475, *n* = 18, *P* < 0.05) and NH_4_^+^(*R* = -0.475, *n* = 18, *P* < 0.05).

**FIGURE 8:**
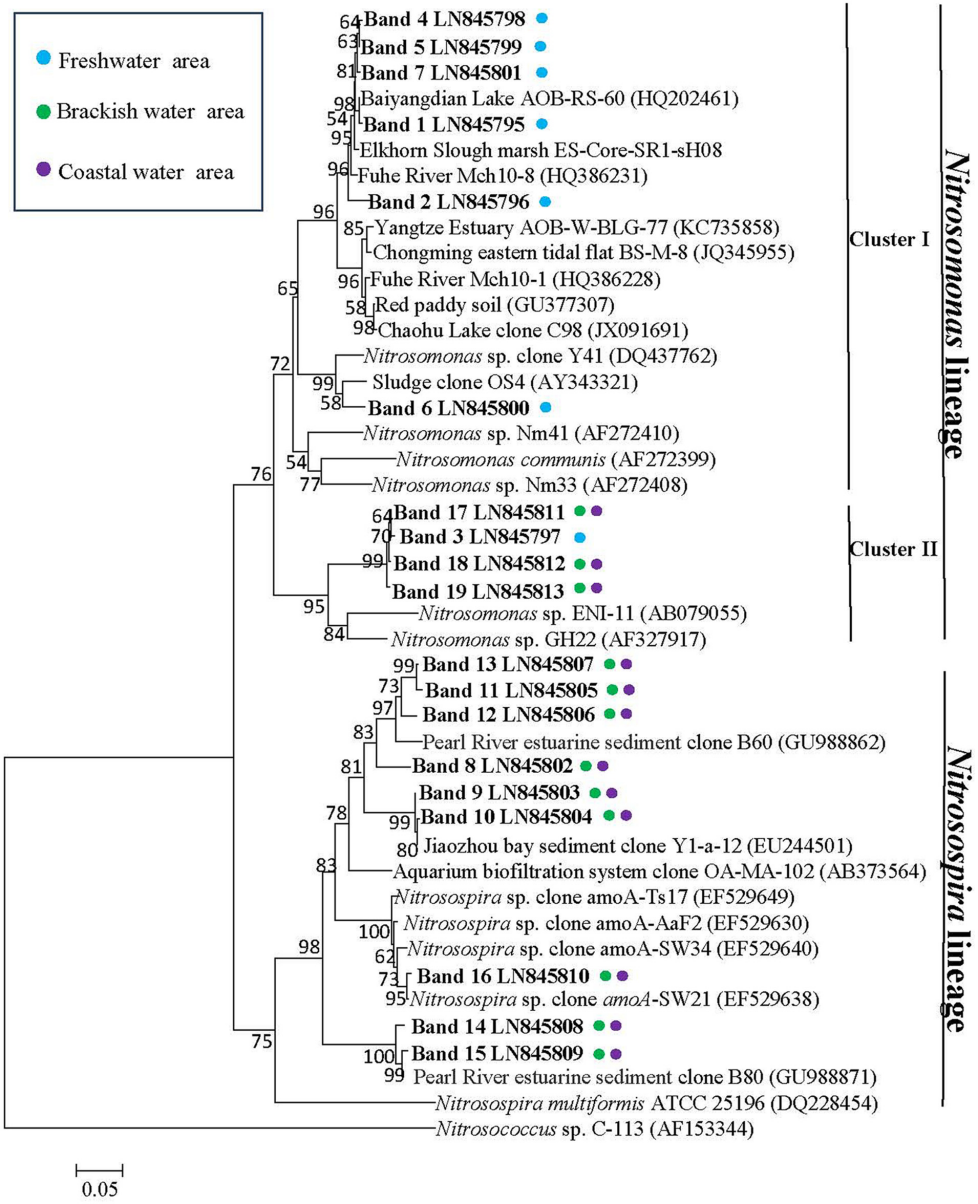
**Phylogenetic relationship among bacterial *amoA* gene sequences retrieved from the surface waters along Yong River.** Designation of the clones in bold includes the following information: accession number in the EMBL with DGGE band label. Colored points next to each retrieved gene sequences indicate the sampling station. Bootstrap values ≥ 50% (1000 neighbor-joining bootstrap) are shown at the branch points. The scale bar represents 5% estimated sequence divergence. The tree was rooted with *Nitrosococcus* sp. C-113 (AF153344).

A total of nineteen bands of the AOB-*amoA* gene were excised from the DGGE gels (**Figure 6**). The recovered sequences (491 bp) were used to build a phylogenetic tree (**Figure 8**). A neighbor-joining analysis indicated that the AOB-*amoA* sequences were grouped into a *Nitrosomonas* and a *Nitrosospira* lineage. The sequences from the freshwater sites fell into the *Nitrosomonas* lineage. Furthermore, the *Nitrosomonas* lineage could be divided into two clusters (I and II). Some of the sequences fell into cluster I, including also *Nitrosomonas* sp. Nm41, *Nitrosomonas communis*, and *Nitrosomonas* sp. Nm33. Sequences distantly related to *Nitrosomonas* sp. ENI-11 and *Nitrosomonas* sp. GH22 grouped into cluster II. With the exception of the sequences of the marine bands 17, 18, and 19, which clustered into *Nitrosomonas* cluster II, the majority of the AOB-*amoA* sequences recovered from W3, W4, W5, and W6 was affiliated with the *Nitrosospira* lineage.

## Discussion

The abundance and composition of AOA*-amoA* and AOB*-amoA* genes in surface waters were determined along the steep physicochemical freshwater to marine water gradient in Ningbo City, China. Clear distinctions among dominant ammonia oxidizers as observed from the DGGE patterns are revealed between freshwater, brackish, and coastal surface waters. Despite the relatively low resolution of DGGE banding patterns, the technique is still ideal as an initial screening method for comparing multiple samples with its low costs and short processing time (Green et al., 2010), provided that the quality of DGGE profile is high enough. Furthermore, when using marker genes DGGE fingerprinting may be an appropriate method to indicate qualitatively and semi-quantitatively the predominant functional microbial groups in ecosystems. It has successfully been applied to study the responses of ammonia monooxygenase (*amoA*) genes to environmental factors in many studies. In our study, clear shifts in predominant AOA and AOB were observed using the PCR-DGGE method and the detected sequences have been analyzed and combined with qPCR data and potential nitrification rates. All these data together showed the responses of the ammonia-oxidizing community on the environmental gradient in eutrophic waters.

### Relative Abundance of AOA and AOB in Relation to Different Environmental Factors

Compared with samples from the brackish (W3 and W4) and coastal (W5 and W6) surface waters, high concentrations of NH_4_^+^-N, NO_2_^-^-N, NO_3_^-^-N, and TOC, and low amounts of DO were observed in samples from the hyper eutrophic waters of W1 and W2 that are situated in long-term stagnant canals (Zhao and Yang, 2009; **Table 2**). The lower *amoA* gene copy numbers of both AOA and AOB in these hyper eutrophic surface waters may be due to one or a combination of these environmental factors.

Ammonia-oxidizing archaea and AOB in surface waters were strongly impacted by salinity. In contrast to the AOB-*amoA* gene abundances, AOA-*amoA* gene numbers in Yong River varied markedly between sampling areas. From freshwater to marine sites AOA increased as much as 13–22 times, whereas AOB only increased 1–2 times. Opposite to our observation, Santoro et al. (2008) observed a nearly constant abundance of AOA across an estuarine gradient at Huntington Beach, while the abundance of AOB was dramatically lower in the freshwater stations compared with saline stations. Bernhard et al. (2007) detected lowest numbers of AOB at the low salinity site of the Plum Island Sound estuary. In a subsequent study of AOA in the same estuary, Bernhard et al. (2010) observed the highest abundance of AOA at the high salinity site in April, but at intermediate salinities in August/September. Such differences between AOA and AOB abundances are reflected in the AOA/AOB ratios. In our study, the ratios of AOA/AOB in W1 (14.74), W2 (9.98), and W3 (3.80) were significantly lower than in W4 (191.88), W5 (143.71), and W6 (153.43) (**Table 3**), which is in contrast with the AOA/AOB ratios that were reported for Plum Island Sound (Bernhard et al., 2010) San Francisco Bay (Mosier and Francis, 2008) and Huntington Beach (Santoro et al., 2008). At these other locations, the relative numbers of AOA decreased with increasing salinity. The difference between the Yong River and the other estuaries may be related to the physical environment, which was the water column for Yong River and sediments for the other studies.

The ratio of AOA/AOB declined with increasing ammonium concentrations. In general AOB dominate at higher ammonium concentrations as shown in a simulated creek ecosystem (Herrmann et al., 2011), lakes (Vissers et al., 2013; Bollmann et al., 2014), wetlands (Sims et al., 2012) and soils (Verhamme et al., 2011), and in the ammonia-rich area of Sacramento River (Damashek et al., 2015). Therefore, our findings support the idea that AOA are more adapted than AOB to oligotrophic environments (Martens-Habbena et al., 2009).

### Potential Niche Differentiation between AOA and AOB

To estimate the ammonia oxidation capacity of the ammonia-oxidizing microorganisms, the potential ammonia-oxidizing activity was determined as described before (Kasuga et al., 2010). Since the method of Kasuga et al. (2010) uses ammonium consumption rates and not nitrite production rates for measuring potential ammonia oxidation, some of the ammonium may have been used by heterotrophic bacteria. However, in activated sludge systems only 10–20% of the ammonium was allocated to ammonium removal by heterotrophic organisms (cited from Kasuga et al., 2010). Potential ammonia oxidation rates do not represent *in situ* rates, but give rather sizes of the active ammonia-oxidizing microbial community and hence can be used for comparison between sites (see also Damashek et al., 2015). The data indicated that PAAs in the freshwater samples (W1 and W2) were lower than those measured in the brackish and coastal water samples, which was different from previous studies that showed increased nitrification activities in freshwater samples compared to more marine environments (Seitzinger, 1988; Bollmann and Laanbroek, 2002; Bernhard et al., 2005). This can be due to the hyper eutrophic conditions and lower DO concentrations (Rysgaard et al., 1994; Liikanen and Martikainen, 2003) in the freshwater part of the Yong River estuary that may inhibit the ammonia oxidation.

The PAA was positively correlated with AOA abundance, but not with AOB abundance, which was consistent with previous studies (Caffrey et al., 2007; Lam et al., 2007). Such a correlation between AOA abundance and PAA, could suggest that AOA were the most active contributors of ammonia oxidation in the surface waters in Yong River. The potential of AOA involved in nitrogen removal was already reported by previous studies (Herrmann et al., 2008; You et al., 2009). However, some studies supported that AOB were the major contributors to ammonia oxidation (Jia and Conrad, 2009; Zhang et al., 2010), especially in high-ammonium habitats where AOB were quantitatively dominant (Wells et al., 2009; Limpiyakorn et al., 2011), and where the potential nitrification activity was positively correlated with AOB, but not with AOA (Herrmann et al., 2011).

### Composition of AOA and AOB Communities in Relation to Environmental Factors

A distinct difference in the composition of the AOA and AOB communities was observed between freshwater on one site and brackish or coastal areas on the other site (**Figures 5** and **8**). It suggested that ammonia oxidizers community composition is shaped by environmental factors. A relationship between *amoA* composition and ecological niches was described in previous studies (Biller et al., 2012; Fernàndez-Guerra and Casamayor, 2012; Hatzenpichler, 2012; Cao et al., 2013).

Within the estuary, a relatively high similarity of AOA community composition was observed between the brackish (W3 and W4) and coastal (W5 and W6) areas (**Figures 3** and **4**). The similarity in community composition between the brackish and marine areas can be due to the intrusion of coastal water, creating similar estuarine environments. A comparable AOA community composition was found at the mouth of the Changjiang Estuary, which flows into the East China Sea approximately 150 km north of Ningbo City (Dang et al., 2008).

However, a clear shift of AOA composition was observed from freshwater to brackish and marine waters. Salinity was considered the most important factor affecting AOA community structure based on *amoA* sequence clustering in aquatic habitats (Cao et al., 2013; Xie et al., 2014). Other environmental factors such as oxygen (Molina et al., 2010; Biller et al., 2012, ammonium concentration (Verhamme et al., 2011) and pH (Nicol et al., 2008) could also contribute to the change in AOA community composition.

The AOA-*amoA* DNA sequences derived from Bands 10 to 21 of DGGE gel, which were obtained from brackish and coastal water samples were highly similar to those from an aquarium system (Sauder et al., 2011), the Changjiang estuary (Dang et al., 2008), the East China Sea (Hu et al., 2011), a wastewater treatment (Bai et al., 2012), hot springs (Zhang et al., 2008; Jiang et al., 2010), the Elkhorn Slough estuary (Wankel et al., 2011), the Pearl River estuary (Jin et al., 2011). The AOA-*amoA* sequences selected from Bands 1 to 9 obtained from freshwater had a high similarity to those collected from a wastewater treatment (Bai et al., 2012), hot spring (Zhang et al., 2008; Jiang et al., 2010), the Elkhorn Slough estuary (Wankel et al., 2011), fresh-water aquaria (Sauder et al., 2011), the Changjiang estuary (Dang et al., 2008), the paddy soil (Fujii et al., 2010), temperate forest soil (Onadera et al., 2010) and a littoral wetland zone at Baiyangdian Lake (Wang et al., 2012).

A relatively high similarity in AOB community was also found between the brackish and coastal areas (**Figures 6** and **7**). However, the Shannon diversity indices of the AOB communities in the brackish and coastal areas were significantly higher than those at calculated from the freshwater stations, which was in contrast with a previous study that showed the loss of diversity of AOB with increasing salinity in Plum Island Sound estuary (Bernhard et al., 2005). The collected sequences of *Nitrosomonas* clustered to sequences obtained from the Yangtze Estuary (Zheng et al., 2014), the Chongming eastern tidal flat (Zheng et al., 2013), a wastewater treatment plant (Gao et al., 2014), a paddy field soil (GenBank unpublished), and Dianchi Lake (GenBank unpublished). One of the *Nitrosospira-*related sequences from the marine area showed 100% identity with sequences from the Chongming eastern tidal flat (Zheng et al., 2013) and the Jiaozhou Bay (Dang et al., 2010), and 99% identity with sequences from the Chongming eastern tidal (Zheng et al., 2013), the Yangtze Estuary (Zheng et al., 2014), red soil (GenBank unpublished) and the Changjiang Estuary (GenBank unpublished), respectively.

*Nitrosomonas*-related sequences were predominant in the freshwater part of the Yong River, whereas *Nitrosospira*-associated sequences came to the fore in the more saline parts of the estuary (**Figure 8**). A shift from a *Nitrosomonas*-dominated community to a *Nitrosospira*-dominated community has been observed before in the Scheldt River when the estuary changes from fresh to brackish to marine conditions (de Bie et al., 2001). From a study of ammonia oxidizers at Huntington Beach, it was suggested that changes in the amount of DO caused the shift in the ammonia oxidizer community (Santoro et al., 2008). However, the ammonia-oxidizing bacterial community in the river Scheldt was impacted by salt rather than by oxygen (Bollmann and Laanbroek, 2002). Organic and nutrient loads of wastewater shaped significantly the AOB community structure by in the eutrophic Tokyo Bay (Urakawa et al., 2006). In our study on the hyper eutrophic Yong River, the composition of the AOB communities at freshwater sites was dissimilar from those at the brackish and marine sampling locations. The freshwater sites were characterized by relatively high concentrations of nitrogen and low salinities and concentrations of oxygen. Hence, it seems likely that these factors govern the structure of the AOB communities leading from predominance of *Nitrosomonas*-related sequences in the nitrogen-rich, but oxygen-limited freshwater parts to predominance *Nitrosospira*-dominated communities in the nitrogen-poor, but more oxygen-rich, saline waters. Therefore, our study suggests that salinity is not the main contributing factor in determining AOB diversity in the low saline regions. Perhaps in more oligotrophic waters, salinity is the most important determinant for diversity of the AOB community composition, but in the hypereutrophic waters, other factors, such as DO, or levels of nitrogen and organic matter become more important, especially in the urban canals of the Yong River system where the surface waters are retained for a long period.

## Conclusion

A clear shift in ammonia-oxidizing activity was observed along the estuary of the River Yong with lower activities in the freshwater, urban zones. Simultaneously with the shift in ammonia-oxidizing activity, shifts in the abundance and species composition of both the archaeal and bacterial ammonia oxidizers were observed together with an increase in the AOA/AOB ratio with increasing salinity. The shifts in activity, abundance and species composition were accompanied by shifts in ammonium and oxygen availabilities, which suggest that these factors together with salinity might impact the characteristics of the ammonia-oxidizing microbial communities. The difference in behavior between AOA and AOB along the urban, estuarine gradient might underlines the physiological differences between these groups. Hence, a better understanding of the nitrogen-converting processes in such highly eutrophic ecosystems is depending on a further exploration of the physiological abilities of AOA and AOB.

## Conflict of Interest Statement

The authors declare that the research was conducted in the absence of any commercial or financial relationships that could be construed as a potential conflict of interest.
